# Accounting for Space — Quantification of Cell-To-Cell Transmission Kinetics Using Virus Dynamics Models

**DOI:** 10.3390/v10040200

**Published:** 2018-04-17

**Authors:** Peter Kumberger, Karina Durso-Cain, Susan L. Uprichard, Harel Dahari, Frederik Graw

**Affiliations:** 1 Center for Modeling and Simulation in the Biosciences, BioQuant-Center, Heidelberg University, 69120 Heidelberg, Germany; peter.kumberger@bioquant.uni-heidelberg.de; 2 Department of Microbiology and Immunology, Loyola University Medical Center, Maywood, IL 60153, USA; kdurso@luc.edu (K.D.-C.), suprichard@luc.edu (S.L.U.); 3 The Program for Experimental and Theoretical Modeling, Division of Hepatology, Department of Medicine, Loyola University Medical Center, Maywood, IL 60153, USA; hdahari@luc.edu

**Keywords:** cell-to-cell transmission, virus dynamics, mathematical modeling, spatial effects

## Abstract

Mathematical models based on ordinary differential equations (ODE) that describe the population dynamics of viruses and infected cells have been an essential tool to characterize and quantify viral infection dynamics. Although an important aspect of viral infection is the dynamics of viral spread, which includes transmission by cell-free virions and direct cell-to-cell transmission, models used so far ignored cell-to-cell transmission completely, or accounted for this process by simple mass-action kinetics between infected and uninfected cells. In this study, we show that the simple mass-action approach falls short when describing viral spread in a spatially-defined environment. Using simulated data, we present a model extension that allows correct quantification of cell-to-cell transmission dynamics within a monolayer of cells. By considering the decreasing proportion of cells that can contribute to cell-to-cell spread with progressing infection, our extension accounts for the transmission dynamics on a single cell level while still remaining applicable to standard population-based experimental measurements. While the ability to infer the proportion of cells infected by either of the transmission modes depends on the viral diffusion rate, the improved estimates obtained using our novel approach emphasize the need to correctly account for spatial aspects when analyzing viral spread.

## 1. Introduction

The way a pathogen spreads within a host has important implications for the successful establishment and maintenance of an infection, as well as the identification of therapeutic targets. Being cellular parasites, viruses have evolved different mechanisms to invade their corresponding target cells in order to replicate and spread infection. In general, these mechanisms can be categorized by two main modes of viral transmission, i.e., (i) cell-free (CF) spread by diffusing viral particles and (ii) direct cell-to-cell (CC) transmission between infected and uninfected cells [1]. While cell-free diffusion of viral particles allows the infection of distant cells, direct cell-to-cell transmission leads to local spread, avoids complex entry processes and is thought to have the potential to shield the virus from neutralizing antibodies and other antiviral clearance mechanisms [2,3]. Therefore, the latter transmission mode is usually thought to be more efficient in spreading infection [4,5]. In addition, CC is assumed to play an important role in the establishment of persistent infections and therapy failure [2,6,7]. The importance and contribution of each of the transmission modes to viral spread still has to be determined in detail. However, to address this question, a reliable quantification of the individual transmission kinetics is needed.

Mathematical models have been an essential tool to analyze experimental data to assess viral infection dynamics. They have allowed the quantification of viral kinetics and identification of potential antiviral therapies (reviewed in [8,9,10]). These virus dynamics models are usually based on systems of ordinary differential equations (ODE) describing the change in concentrations of target and infected cells, as well as the viral load over time. The earliest models, including the so-called standard model of virus dynamics [8], concentrated on cell-free transmission describing the spread of infection by a contact process between virions and target cells [11,12]. Mathematically, this transmission was formulated by simple mass-action kinetics. While these models provided first estimates of viral infection kinetics and cellular turnover dynamics, they ignored the process of cell-to-cell transmission. With increasing awareness of the importance of cell-to-cell transmission for infection dynamics, revised models appeared that additionally accounted for this transmission mode by extending the standard model of virus dynamics [13,14,15,16,17]. These new models considered different specific aspects of cell-associated transmission, including the possibility to transfer multiple viral strains simultaneously [16,17], as well as considering a time-delay for viral production [13]. However, all these models described cell-to-cell transmission by allowing target cells to get infected proportional to the concentration of infected cells and assumed that target and infected cells are well mixed. This assumption is especially violated for viral spread among stationary cells, e.g., within solid tissue environments. As the infection progresses, infected cells successively become surrounded by other infected cells, hindering cells in the center of infected foci from contributing to cell-to-cell transmission. Such foci of infected cells have been observed, for example, in HCV-infected liver tissue [18,19,20]. Thus, previous models relying on the mass-action assumption overestimate the fraction of infected cells contributing to cell-to-cell transmission and lead to an inappropriate quantification of the infection dynamics. There have been several approaches to improve these quantifications for various pathogens by using probabilistic [21] and spatially explicit modeling frameworks, such as agent-based models [22,23]. However, especially the latter approach is difficult to be directly fit to experimental data. Therefore, finding an extension for the standard model of virus dynamics that appropriately accounts for cell-to-cell transmission would help to improve current analyses of viral spread.

To this end, we developed an agent-based model (ABM) to simulate viral replication and spread in a monolayer of cells. The simulated data allowed us to reveal the deficiencies of standard virus dynamics models when analyzing viral spread by cell-to-cell transmission. Based on these analyses, we developed an extension for the standard model of virus dynamics to allow for an appropriate quantification of the infection kinetics due to cell-to-cell transmission. Our novel approach adjusts for the decreasing proportion of infected cells contributing to cell-to-cell spread with progressing infection, which is especially important for viral transmission among stationary cells. In contrast to current models, our proposed model provides a more accurate quantification of the underlying infection dynamics. As such, our extension provides a means to account for viral transmission dynamics on a single cell level within the context of a model that remains applicable to common population-based experimental measurements that are used to quantify rates characterizing viral infection and spread. Additionally, we tested the ability of our novel method in comparison to previous approaches to infer the contribution of each of the transmission modes to infection spread and found that reliable approximations of these contributions depend on the diffusion rates of cell-free virus, with our novel approach leading to more appropriate estimates. Thus, our analysis provides an improved understanding of how to reliably quantify cell-to-cell transmission dynamics for various viruses spreading within solid tissue-environments.

## 2. Materials and Methods

### 2.1. The Standard Model of Viral Dynamics

The standard model of virus dynamics describes the coupled dynamics of the concentration of target cells, *T*, infected cells, *I*, and the extracellular viral load, *V* [8]. In general, target cells are assumed to get infected at rate $\beta_{f}$ proportional to the viral concentration *V* and have an average lifetime of $1/\delta_{T}$. Infected cells produce new virions with a viral production rate ρ and are lost with rate $\delta_{I}$, while free virions are cleared with rate *c*. Previous model extensions accounted for cell-to-cell transmission by including an additional infection term, also allowing target cells to get infected at a rate $\beta_{c}$ proportional to the concentration of infected cells [13,14,15]. Hereby, $\beta_{c}$ describes the rate of cell-to-cell transmission. In summary, the basic model accounting for both transmission modes is then described by the following system of ordinary differential equations:

(1) $$\begin{array}{cc} \frac{dT}{dt} & =-\beta_{f}VT-\beta_{c}IT-\delta_{T}T\\ \frac{dI}{dt} & =\beta_{f}VT+\beta_{c}IT-\delta_{I}I\\ \frac{dV}{dt} & =\rho I-cV. \end{array}$$

As we will later introduce different extensions and sub-models of this standard model, Table 1 provides an overview of all the different models used in the current study.

### 2.2. Simulating Viral Spread in a 2D Agent-Based Model

We developed and simulated spread of a positive-strand RNA virus within a monolayer of cells in vitro using an agent-based modeling approach. Cells were distributed on a two-dimensional lattice with each node denoting a single cell. We assume that each cell has a hexagonal shape with k=6 direct neighbors and the total hexagonal shaped grid comprising 24,031 cells in total (90 cells per side).

**Figure 1:**
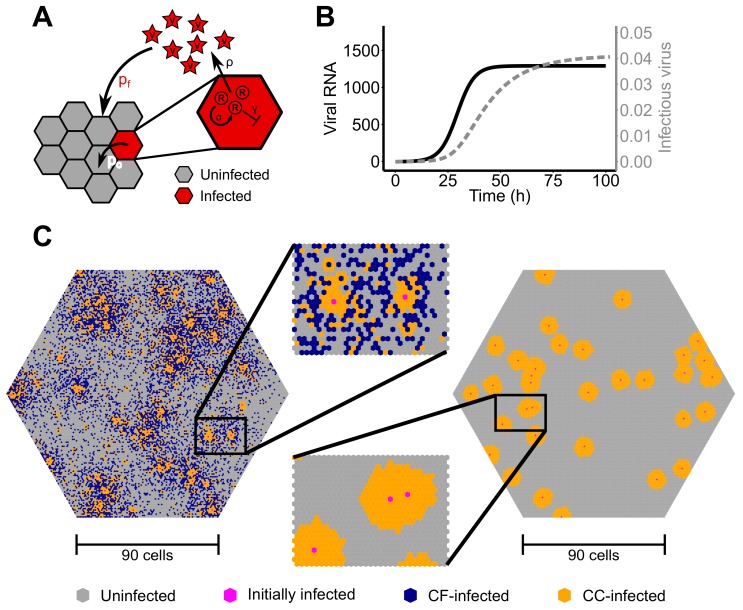
(**A**) Schematic of the agent-based model simulating intracellular viral replication and spread by cell-free (CF) and cell-to-cell (CC) transmission among stationary cells. Cells are assumed to be hexagonally shaped and can be either uninfected (gray) or infected (red). The parameters $p_{c}$ and $p_{f}$ define the probability of infection by CC- and CF-transmission, respectively, dependent on the intra- and extra-cellular viral load at the corresponding grid sites; (**B**) Simulated time courses of intracellular viral load (black line) and produced extracellular virus (gray line) for one infected cell; (**C**) Realization of simulation outcomes after around three days post infection assuming simultaneous occurrence of CF- and CC-transmission (left) or only CC-transmission (right). Cells infected by CF or CC-transmission are indicated in blue and orange, respectively.

A sketch of the different processes considered in the agent-based model is depicted in Figure 1A. Cells are stationary and can be either infected or uninfected. Upon infection of a cell, intracellular viral replication is modeled by an ordinary differential equation describing the accumulation of positive-strand RNA, *R*. We assume density dependent replication with a maximal replication rate α and a carrying capacity of $R_{\mathrm{cap}}$ for each cell. Positive-strand RNA is degraded with rate γ and exported from the cell with an export rate ρ contributing to the extracellular viral concentration, *V*. However, only a fraction of the intracellular viral RNA, $f_{\inf}$, is assumed to be infectious. Thus, the intracellular replication of positive-strand RNA in an infected cell at site (i,j) and its export is described by the following system of ordinary differential equations:

(2) $$\begin{array}{cc} \frac{dR_{i,j}}{dt} & =\alpha R_{i,j}\left(1-\frac{R_{i,j}}{R_{\mathrm{cap}}}\right)-\gamma R_{i,j}-\rho R_{i,j}\\ \frac{dV_{i,j}}{dt} & =f_{\inf}\rho R_{i,j}-cV_{i,j}. \end{array}$$

Extracellular virus is capable of diffusing through the lattice with diffusion modeled as seen in [24] assuming that the viral concentration at grid site $\left(i_{0},j_{0}\right)$ changes from time step $t_{n}$ to $t_{n+1}$ by:

(3) $$V_{i_{0},j_{0}}\left(t_{n+1}\right)=V_{i_{0},j_{0}}\left(t_{n}\right)-\frac{m}{k}\sum_{(i,j)\in \Omega }\left(V_{i,j}\left(t_{n}\right)-V_{i_{0},j_{0}}\left(t_{n}\right)\right),$$

with *k* and Ω denoting the number and set of neighboring grid sites, respectively, and *m* the fraction of viral particles that are assumed to diffuse. An uninfected cell can get infected by cell-free transmission at each time-step with probability $p_{f}$ dependent on the extracellular viral concentration at the corresponding grid site, *V*, and a scaling factor $s_{f}$, i.e., $p_{f}=s_{f}V$. Cell-to-cell transmission is considered by the direct transmission of intracellular viral RNA from an infected cell to its uninfected neighbor and occurs with probability $p_{c}$ dependent on the intracellular concentration of infectious viral RNA within the infected cell, $f_{\inf}R$, and a scaling factor $s_{c}$. Upon successful infection, the concentration of intracellular viral RNA within the infecting cell is reduced due to transfer of viral material into the target cell and the novel infected cell starts viral replication with one single viral RNA. Initial viral load for cells infected through cell-free infection is also one viral RNA. Intracellular viral replication is parameterized by fitting Equation (2) to experimental data on the spread of HCV within in vitro cultures that measured the intracellular RNA per cell and the extracellular viral load over time [25]. The estimated parameter values considered within the simulation are shown in Table S1 and the resulting time courses of the concentrations for intracellular RNA, *R*, and extracellular infectious virus, *V*, for one infected cell are shown in Figure 1B.

Resembling previous experimental protocols [21], our ABM-simulated infection is initialized by choosing a number of cells at random that are infected with probability $p^{\mathrm{init}}\left(t\right)$ during the first 17 h of a simulation. Hereby, $p^{\mathrm{init}}\left(t\right)$ is defined by:

(4) $$p^{\mathrm{init}}\left(t\right):=\hat{I_{0}}\frac{\lambda e^{-\lambda t}}{1-e^{-17\lambda }}\;\;,$$

with $\hat{I_{0}}$ denoting the expected total number of infected cells during initialization, and λ the rate at which the inoculum used for infection looses its infectivity. At 17 h post infection, the total extracellular virus concentration is reset to zero, representing the change of media.

The simulated cell culture system was run for 10 days and the number of infected cells, as well as the viral concentration at indicated time points was noted. The appropriateness of different population-based modeling approaches to infer the underlying parameters characterizing both transmission modes was determined by fitting these models to the simulated ABM-data. The probabilities for cell-free, $p_{f}$, and cell-to-cell transmission, $p_{c}$, were varied between simulations to consider different contributions of each of the two transmission modes to infection spread. An example of a simulation of the agent-based model about 3 days after the initiation of infection assuming a scaling factor $s_{f}=28\;\min^{-1}\;{\mathrm{virus}}^{-1}$ and $s_{c}=0.82\;\min^{-1}\;{\mathrm{RNA}}^{-1}$ is shown in Figure 1C. The model was implemented in the C++ programming language.

### 2.3. Parameter Estimation

The different mathematical models describing the spread of infection, e.g., Equation (1), were fitted to the simulated data using the optim-function in the *R*-language of statistical computing [26]. Parameters characterizing the infection dynamics were determined maximizing the log-likelihood function given by:

(5) $$\begin{array}{cc} l(\theta )= & -\frac{1}{2}\sum_{t=t_{0}}^{t_{m}}{\left(\frac{\bar{x}\left(t\right)-f\left(\theta ,t\right)}{\sigma_{t}}\right)}^{2}-\frac{n}{2}\ln\sigma^{2},\;\;\mathrm{with} \end{array}$$

(6) $$\begin{array}{cc} \sigma^{2}= & \frac{\sigma_{t}^{2}}{n}+\sigma_{\mathrm{me}}^{2}\;\;\;\mathrm{and}\;\;\;\bar{x}\left(t\right):=\frac{1}{n}\sum_{j=1}^{n}x_{j}\left(t\right) \end{array}$$

Hereby, *n* determines the number of different simulations, $x_{j}\left(t\right)$ the measured concentration of infected cells or the viral concentration at sampling time point *t* for simulation *j*, $\sigma_{t}$ the empirical variation across all simulations, and f(θ,t) the corresponding model prediction with $\theta =(\beta_{c},\beta_{f},\ldots )$. Furthermore, we assume a constant absolute measurement error, $\sigma_{\mathrm{me}}$, that was additionally estimated. When the extracellular viral concentration and the number of infected cells were considered in the fit, a relative measurement error was assumed. Parameter identifiability was assessed using a profile likelihood approach [27], which was also used to calculate the 95%-confidence intervals.

Model performance was compared using the corrected Akaike information criterion (AICc) [28] calculated by:

$$\mathrm{AICc}=2k-2L+\frac{2k(k+1)}{n-k-1},$$

where L=max(l(θ)) defines the maximal value of the log likelihood function, *k* the number of model parameters and *n* the number of data points the model is fitted to. Differences between models were evaluated by the ΔAICc with the difference always calculated compared to the best performing model with the lowest AICc-value within the corresponding situation.

## 3. Results

### 3.1. Standard Models of Virus Dynamics Are Insufficient to Describe Cell-To-Cell Transmission Dynamics among Stationary Cells

**Figure 2:**
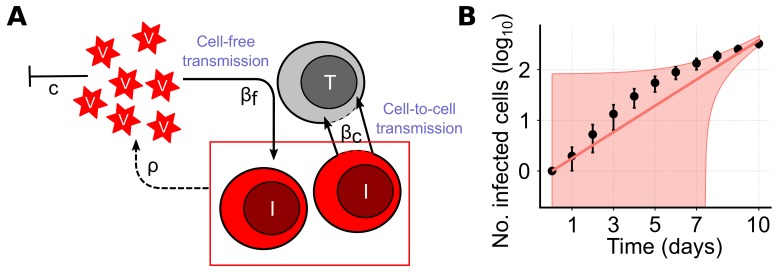
(**A**) Sketch of a viral dynamics model in a well-mixed system as given in Equation (1). In case of cell-to-cell (CC) transmission target cells (*T*, gray) become infected (*I*, red) with rate $\beta_{c}$ dependent on the concentration of target and infected cells. Infection by cell-free virus occurs with rate $\beta_{f}$ dependent on the viral load, *V*, and the target cell concentration. Viral particles are produced by infected cells with rate ρ and cleared at rate *c*. For simplicity, the processes of cell death and proliferation are neglected here; (**B**) Simulated dynamics and model predictions for viral spread only considering CC among stationary cells starting with a single infected cell. Black dots indicate the mean and vertical lines represent 95%-confidence interval for the total number of infected cells averaged over 100 individual ABM simulations sampled every 24 h. The red line indicates the best fit of the basic model as described in Equation (1) with $\beta_{f}=\delta_{I}=\delta_{T}=0$ to the data, with the red shaded area indicating the 95%-confidence interval for model predictions using the estimated standard deviation.

The standard model of virus dynamics has been extensively used to analyze time courses of infection. It describes the dynamics of the concentration of target cells, *T*, infected cells, *I*, and the extracellular viral load, *V*, accounting for the infection processes and viral turnover [8] (see Figure 2A and Equation (1) in the Materials and Methods for a detailed description of the model). In this model, the two infection processes of CF- and CC-transmission are generally described by mass-action kinetics dependent on the viral concentration, *V*, and the concentration of infected cells, *I*. Examining the latter term addressing cell-to-cell transmission in more detail, it becomes clear that all infected cells are assumed to always contribute to CC-transmission. However, this assumption is most likely violated for viral spread within spatially defined environments, especially for viral spread among stationary cells, as, e.g., in solid tissues.

To test the ability of the basic model defined in Equation (1) to describe cell-to-cell transmission dynamics among stationary cells, we used an agent-based model (ABM) simulating viral spread in a 2D monolayer of cells. The ABM assumes cells to be distributed in a hexagonal grid, i.e., with each cell having six different neighbors, and accounts for intracellular viral replication and intercellular viral spread (see the Materials and Methods for a detailed explanation). For simplicity, proliferation and death of infected and uninfected cells were neglected. To concentrate on the dynamics of cell-to-cell transmission, we first studied infection spread by cell-to-cell transmission starting with a single infected and assuming unlimited target cell numbers. Figure 2B shows the increase in the total number of infected cells averaged over 100 individual ABM simulations that were evaluated every 24 h for 10 days. Fitting a model that assumes standard mass-action kinetics to describe cell-to-cell transmission (CC-model, Equation (1) with $\beta_{f}=\delta_{I}=\delta_{T}=0$) to these data, we find that it is incapable of providing a reasonable representation of the infection dynamics (Figure 2B, with reduced Chi-squared-statistics $X_{8}^{2}=1096$ indicating poor fit considering the standard deviation of the data). Such a model fails to explain the observed decreasing growth rate of a focus of infected cells over time. Thus, simple mass-action kinetics as it has been used previously [11,13,14] seems to be inappropriate when describing viral spread by cell-to-cell transmission among stationary cells.

### 3.2. Improved Description of Cell-To-Cell Transmission Dynamics Accounting for Spatial Effects

**Figure 3:**
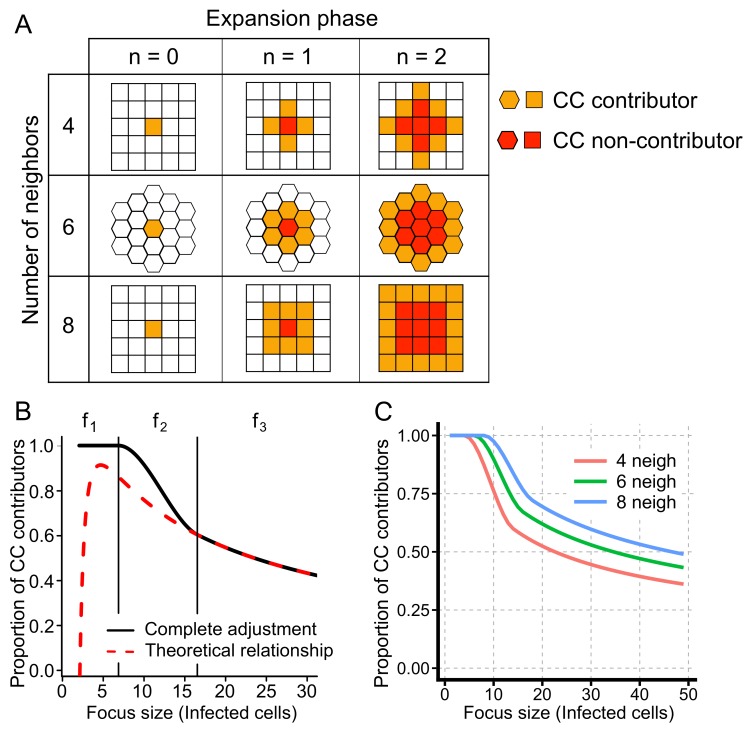
Proportion of cells contributing to cell-to-cell (CC) transmission. (**A**) Sketch of cells contributing to focus growth for different numbers of neighbors per cell and different expansion phases assuming radial focus growth. Whereas cells in the perimeter of the focus (orange) can contribute to CC-transmission, cells completely enclosed by other infected cells (red) cannot; (**B**) Proportion of cells contributing to CC-transmission dependent on the focus size assuming that each cell has six neighbors (k=6). The theoretically-determined relationship (Equation (9), red dashed line) underestimates the true proportion for small focus sizes. The black line indicates the complete adjustment term as described in Equation (10)) with z=10; (**C**) Proportion of CC contributors with respect to focus size assuming either k=4 (red), 6 (green) or 8 (blue) neighbors (neigh) for each cell. Parameter *z* is set to 10.

Our ABM-simulated data indicate a non-constant growth rate for foci of infected cells by cell-to-cell transmission among stationary cells. To better understand the dynamics of focus growth by cell-to-cell transmission over time, we consider a virus only capable of spreading by cell-to-cell transmission. Newly-infected cells can only appear next to already infected cells leading to large foci of infected cells. The larger a focus gets, the larger the proportion of cells that are completely surrounded by other infected cells and, hence, can no longer contribute to cell-to-cell transmission (Figure 3A). Thus, the previously assumed proportionality between successful cell-to-cell transmissions and the concentration of infected cells (Equation (1)) might not hold true and needs to be adapted.

To this end, we derived a correction term $f_{c}\left(I\right)$ that adjusts the infection term $\beta_{c}IT$ for the decreasing proportion of cells contributing to cell-to-cell transmission during the progression of infection. Following a single focus of infected cells in a 2D layer of stationary cells and assuming radial focus growth, i.e., all cells in a certain radius around the focus-founding cell are infected before the next expansion phase (Figure 3A), the proportion of cells able to contribute to cell-to-cell transmission, $f_{c}$, can be calculated by the ratio between the number of cells in the perimeter of the focus and the total focus size,

$$f_{c}=\frac{\#\mathrm{cells}\;\mathrm{in}\;\mathrm{perimeter}\;\mathrm{of}\;\mathrm{focus}}{\#\mathrm{cells}\;\mathrm{in}\;\mathrm{focus}}.$$

The number of cells in the perimeter depends on the assumed number of neighbors per cell, *k*, and the radius of infection, i.e., the expansion phase, *n* (Figure 3A). Initially, having only one infected cell, $f_{c}=1$ as the perimeter equals the whole focus size. After the first expansion phase, n=1, the number of cells in the perimeter is equivalent to the total number of neighbors of a cell, i.e., $f_{c}=k$. After the second and third expansion phase, the number of cells in the perimeter doubles and triples, respectively, while the total focus size grows slightly faster (Figure 3A). In general, the proportion of cells in a focus contributing to cell-to-cell transmission (CC contributors) after completion of expansion phase *n* and dependent on the assumed number of neighbors, *k*, is determined by:

(7) $$\begin{array}{cc} f_{c}\left(n,k\right)=\frac{kn}{1+k\sum_{i=1}^{n}i}=\frac{2kn}{kn^{2}+kn+2} & \;\mathrm{for}\;n\geq 1. \end{array}$$

Similarly, the total number of infected cells in a focus is calculated by:

(8) $$I\left(n,k\right)=\frac{k}{2}n^{2}+\frac{k}{2}n+1.$$

By solving Equation (8) for *n* and substituting in Equation (7), we obtain:

(9) $$f_{c}\left(k,I\right)=\frac{\sqrt{k^{2}+8kI-8k}-k}{2I}$$

as a continuous function of the assumed number of neighbors per cell and the number of infected cells in the focus (see Figure 3B, dashed red line).

Obviously, Equation (9) only holds true if I≥k. Otherwise, all infected cells in the focus can still contribute to cell-to-cell transmission as not all neighbors of the founder cell are infected (see Figure 3A). To allow for a better approximation of the proportion of contributors for small foci sizes, we define $f_{c}\left(I\right)=f_{1}=1$ for I≤k. In order to obtain a smooth function for $f_{c}$ dependent on the number of infected cells, we define a connection term $f_{2}$ for k<I<k+z, with $z\in R^{+}$, to connect the proportion of contributors for small focus sizes, $f_{1}$, with the general function for larger focus sizes obtained in Equation (7) (see Figure 3B and Supplementary Material Text S1 for a detailed derivation of the connection term $f_{2}$). Hereby, the parameter *z* defines the number of infected cells over which the transition between full contributors and ringlike focus growth is used. Thus, the final adjustment term $f_{c}\left(I\right)$ for a single focus comprising *I* infected cells is then given by:

(10) $$\begin{array}{c} f_{c}\left(I\right)=\left\{\begin{array}{cc} 1 & \mathrm{for}\;I\leq k\\ a{\left(I-k\right)}^{3}+b{\left(I-k\right)}^{2}+1 & \mathrm{for}\;k<I<k+z\\ \frac{\sqrt{k^{2}+8kI-8k}-k}{2I} & \mathrm{for}\;I\geq k+z, \end{array}\right. \end{array}$$

where a=a(z,k) and b=b(z,k) are functions of the parameters *z* and *k* and define coefficients of the polynomial for $f_{2}$ ensuring the smoothness of $f_{c}$ (see Text S1). Figure 3C shows the development of the proportion of CC contributors for different numbers of neighbors per cell dependent on the focus size, *I*. Note that the adjustment term described in Equation (10) is valid for a continuous variable *I*, e.g., *I* representing the concentration of infected cells. To approximate the proportion of cells contributing to cell-to-cell transmission in a culture comprising several foci, *I* can be replaced by I/ϕ, where *I* defines the total number of infected cells and ϕ the number of different foci at the beginning.

With the definition of $f_{c}$, an adjusted model for cell-to-cell transmission dynamics that extends Equation (1) is then defined by:

(11) $$\begin{array}{ccc} \frac{dT}{dt} & =-\beta_{f}VT-f_{c}\left(I\right)\beta_{c}IT-\delta_{T}T\\ \frac{dI}{dt} & =\beta_{f}VT+f_{c}\left(I\right)\beta_{c}IT-\delta_{I}I\\ \frac{dV}{dt} & =\rho I-cV & . \end{array}$$

As we always neglect cellular turnover in our simulations to focus on the effect of the spatial conditions on the transmission processes, cell death is neglected in the following, meaning $\delta_{T}=\delta_{I}=0$ throughout the manuscript.

### 3.3. Adjusted Model Provides Improved Description of Cell-To-Cell Transmission Dynamics for Individual Focus Growth

**Figure 4:**
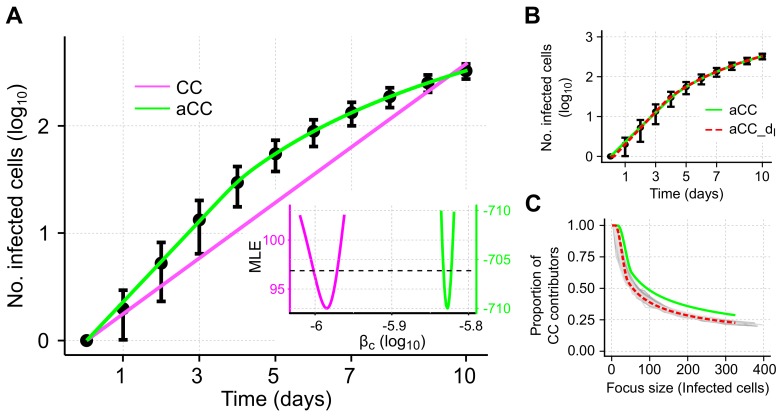
Simulated data and corresponding model predictions describing growth of an individual focus of infected cells. (**A**) Individual focus growth by cell-to-cell (CC) transmission among stationary cells starting with a single infected cell. Black dots indicate the mean and 95%-confidence interval for the total number of infected cells averaged over 100 individual ABM simulations that were sampled every 24 h. Model predictions using standard mass-action kinetics (CC-model, Equation (1) with $\beta_{f}=0$, magenta) or our adjusted model (aCC-model, Equation (11) with $\beta_{f}=0$, green) to describe cell-to-cell transmission dynamics. Insertion: Profile likelihoods of the estimated cell-to-cell transmission rate, $\beta_{c}$, using either the CC- (magenta) or aCC- (green) model; (**B**) An additional model extension considering a time-delay for the time infected cells need to become infectious (aCC-$d_{I}$, red) additionally improves model predictions; (**C**) Proportion of CC contributors as predicted by the aCC-model (green line), and the extended aCC-$d_{I}$-model (red dashed line) in comparison to the actual proportion observed in the ABM simulations (gray lines). The corresponding parameter estimates are given in Table 2.

To test the ability of our adjusted model to describe individual focus growth by cell-to-cell transmission, we fit it to the same simulated data. The adjusted model (aCC-model, Equation (11) with $\beta_{f}=\delta_{T}=\delta_{I}=0$, see also Table 1) provides a visibly better description of the growth dynamics than the standard CC-model (Figure 4A). Model fits are significantly improved (ΔAICc=823.1 compared to ΔAICc=29.2 for the CC- and aCC-model, respectively) and with the adjusted cell-to-cell transmission rate, $f_{c}\left(I\right)\beta_{c}$, we are able to explain the decreasing growth rate of a focus over time.

Estimates for the transmission rate characterizing cell-to-cell spread using the aCC-model are roughly 1.5-times larger than for the CC-model ($\beta_{c}=1.49\times 10^{-6}{\mathrm{cell}}^{-1}h^{-1}\;\left[1.47,1.50\right]$ aCC-model vs. $\beta_{c}=1.04\times 10^{-6}{\mathrm{cell}}^{-1}h^{-1}$[1.00,1.07] CC-model, Table 2). Profile likelihood analysis indicates that all parameters are identifiable based on the given data (Figure 4A, insert and Figure S1).

To more closely resemble the simulated scenario, we also considered a model that additionally incorporated a time-delay for the concentration of infected cells mediating cell-to-cell transmission, i.e., accounting for the time that cells need to become infectious. To this end, the term $f_{c}\left(I\right)\beta_{c}IT$ describing cell-to-cell transmission in the aCC-model is replaced by $f_{c}\left(I\right)\beta_{c}I\left(t-\tau \right)T$, with τ defining the considered time-delay. This revised aCC-d${}_{I}$-model further improves model predictions (Figure 4B and ΔAICc=29.2 compared to the aCC-model, Table 2). However, including a time-delay on the standard-model, i.e., CC-d${}_{I}$, does not improve model predictions substantially (ΔAICc=810.9 compared to aCC-d${}_{I}$), indicating that the additional consideration of an eclipse phase does not explain all the dynamics observed in the data.

To validate our theoretically derived adjustment term $f_{c}$, we compared the predicted proportion of cells contributing to cell-to-cell transmission (CC contributors) to the proportions inferred from the individual ABM simulations. We found that the aCC-model predicts a slightly higher proportion of CC contributors dependent on the focus size than observed in the simulations (Figure 4C, green line). However, the aCC-model with an additional time-delay in the concentration of infected cells contributing to CC (aCC-d${}_{I}$-model) leads to nice agreement in the predicted and observed proportions (Figure 4C, red-dotted line).

In summary, our adjusted model is a more appropriate approach than previous models with simple mass-action kinetics when describing individual focus growth among stationary cells. In addition, it provides a good approximation of the proportion of cells capable of contributing to cell-to-cell transmission.

### 3.4. Determining Cell-To-Cell Transmission Dynamics across Multiple Foci

**Figure 5:**
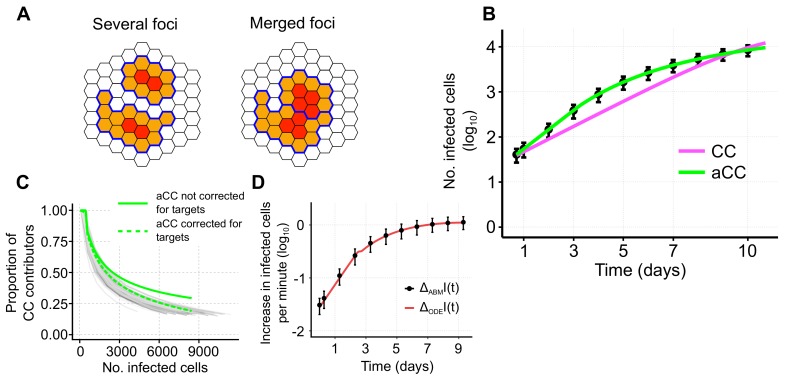
(**A**) Sketch of the same number of infected cells distributed on two foci that are either separate or build one merged focus. Cells contributing to cell-to-cell (CC) transmission (orange) and non-contributors (red) are indicated. Boundaries of separate foci are marked in blue; (**B**) Simulated data and model predictions for a scenario allowing for multiple foci growth. Black dots indicate the mean and 95%-confidence interval for the total number of infected cells averaged over 100 individual ABM simulations that were sampled every 24 h. Colored lines indicate the best fit using either the CC- (magenta) or adjusted aCC-model (green). Corresponding parameter estimates are shown in Table 2; (**C**) Proportion of CC contributors predicted by the adjustment term (green solid line) as defined in Equation (10) in comparison to the proportions inferred from each individual simulation (gray lines). The dashed green line represents the corrected adjustment term multiplied with the proportion of target cells relative to the number of target cells at the beginning of the simulation, i.e., $f_{c}\left(I\left(t\right)\right)T\left(t\right)/T_{0}$; (**D**) Approximate increase of infected cells calculated per minute in the ABM (black dots, mean and 95%-confidence intervals based on 100 simulations) and the ODE model (red line) as defined by Equations (12) and (13), respectively.

Data from in vitro experiments usually comprise the simultaneous growth of multiple foci with measurements of viral load and infected cells obtained on a population level. Here, heterogeneity in the time at which individual foci are initiated, as well as processes such as merging of foci (Figure 5A) might violate the assumptions used for the derivation of the adjustment term $f_{c}$.

To evaluate the appropriateness of our adjusted model to correctly infer cell-to-cell transmission dynamics given the simultaneous growth of multiple foci, we used our agent-based model to simulate viral spread assays that allow the initiation of multiple foci. According to previous experimental conditions [21,29,30], we consider a monolayer of cells in which infection is randomly initiated during a time period of 17 h. After this initial infection period, the extracellular virus concentration is set to zero and cell-free infection is turned off, resembling the removal of medium and the addition of antibodies targeting the viral envelope in the experiment [21,29,30]. Infection is then assumed to progress solely by cell-to-cell transmission. As before, the total number of infected cells in the simulations is sampled every 24 h (see the Materials and Methods for a detailed description of the ABM). To compare the CC- and aCC-model in their ability to describe cell-to-cell transmission dynamics, we separate the two processes of initiation of infection and subsequent focus growth. Therefore, only the data after the end of the initial infection period are considered for the analysis, i.e., taking the number of infected cells 17 h post infection as initial condition.

Again, we observed that our aCC-model performs better than the CC-model that relies on the well-mixed assumption between target and infected cells (Figure 5B and Table 2, $\Delta {\mathrm{AIC}}_{c}=92.88$). In this more complex scenario, a model not accounting for a decreasing growth rate with progression of infection still fails to describe the decelerated infection dynamics. As already observed for a single focus, using the adjusted model (aCC) leads to approximately a 1.5-fold increase in the estimates for the cell-to-cell transmission rate ($\beta_{c}=1.13\times 10^{-6}\;{\mathrm{cell}}^{-1}\;h^{-1}$ [1.07,1.18] CC-model, $\beta_{c}=1.72\times 10^{-6}\;{\mathrm{cell}}^{-1}\;h^{-1}$ [1.70,1.74] aCC-model, Table 2). In addition, we found that estimates of the cell-to-cell transmission rate for the aCC-model are robust even if the assumed neighbor-distribution, *k*, deviates to some extent from the correct distribution (see Supplementary Material Table S2). Extending our adjusted aCC-model to additionally account for the time a cell needs to become infectious (aCC-$d_{I}$) does not improve model fits as we estimate a delay of τ≈0. This is expected as, in contrast to the scenario starting with a single infected cell, there are already some infectious cells present after the end of the initiation period. Therefore, an additional time-delay can be neglected and we will concentrate on the aCC-model for the underlying scenario.

To confirm the suitability of the adjustment term $f_{c}$ in such a complex scenario, we compared the proportion of CC contributors among all foci of infected cells inferred from the ABM simulations to the proportion predicted by our model (Figure 5C). Here, the adjustment seems to provide a worse prediction for the proportion of CC contributors than compared to single focus growth (Figure 4C). Especially for later time points with multiple infected cells, we seem to overestimate the proportion of cells contributing to infection spread. However, the discrepancy between the proportion of CC contributors in the ABM simulations and the predicted proportion by our aCC-model is due to an uneven comparison of both terms. For the ABM, the average increase in infected cells per time step, $\Delta_{\mathrm{ABM}}I\left(t\right)=I\left(t+1\right)-I\left(t\right)$, can be described by:

(12) $$\begin{array}{cc} \Delta_{\mathrm{ABM}}I\left(t\right) & ={\tilde{\beta }}_{c}{\bar{V}}_{cc}{\tilde{f}}_{c}\left(I,T\right)I\left(t\right)\\ \mathrm{with}\;\;\;{\bar{V}}_{cc} & :=\frac{I\left(t_{\mathrm{end}}\right)-I\left(t_{\mathrm{start}}\right)}{{\tilde{\beta }}_{c}\int_{t_{\mathrm{start}}}^{t_{\mathrm{end}}}I\left(s\right){\tilde{f}}_{c}\left(I,T\right)ds}. \end{array}$$

Here, $t_{\mathrm{start}}$ and $t_{\mathrm{end}}$ represent the start and end time of the simulation run, respectively, and ${\bar{V}}_{cc}$ the approximated average concentration of viral particles in the cells contributing to cell-to-cell transmission. The term ${\tilde{\beta }}_{c}$ defines the infectivity per intracellular viral particle, and $f_{c}\left(I,T\right)$ the proportion of infected cells contributing to cell-to-cell transmission by accounting for the availability of uninfected target cells in their neighborhood.

On the other hand, the increase in infected cells in our aCC-model (Equation (11) with $\beta_{f}=\delta_{I}=\delta_{T}=0$) is given by:

(13) $$\Delta_{\mathrm{ODE}}I\left(t\right)=\beta_{c}f_{c}\left(I\left(t\right)\right)\;I\left(t\right)T\left(t\right).$$

Both equations, Equations (12) and (13), describe the same increase, i.e., $\Delta_{\mathrm{ABM}}I\left(t\right)=\Delta_{\mathrm{ODE}}I\left(t\right)$ (Figure 5D) with $\beta_{c}={\tilde{\beta }}_{c}{\bar{V}}_{cc}$. Hereby, the proportion of contributors in Equation (13), $f_{c}\left(I\left(t\right)\right)$, needs to be corrected for the proportion of target cells that are still available (Figure 5C, dashed green line). This is already implicitly considered in the corresponding term ${\tilde{f}}_{c}\left(I,T\right)$ for the ABM. Thus, ${\tilde{f}}_{c}\left(I,T\right)$ corresponds to $f_{c}\left(I\left(t\right)\right)T\left(t\right)/T_{0}$, where $T\left(t\right)/T_{0}$ describes the proportion of available target cells at time *t* with $T_{0}$ defining the total number of target cells at the start of the infection dynamics. In summary, although heterogeneous growth of multiple foci and merging of foci violate the assumptions used to derive the adjustment term $f_{c}$, the term still provides a reliable approximation of the proportion of cells contributing to focus growth (Figure 5C) if corrected for the available proportion of target cells. Therefore, our extended aCC-model provides a better description of the observed infection dynamics than standard models when analyzing viral spread by cell-to-cell transmission among stationary cells.

### 3.5. Using Population Dynamics Models to Disentangle Combined Transmission Dynamics

So far, we have only considered the spread of infection by cell-to-cell transmission and showed that the adjusted CC model (aCC) provides a good description of the underlying dynamics. However, during infection, both transmission modes most likely occur simultaneously. This leads to the question to which extent population dynamics models, as described by Equations (1) and (11), are generally able to disentangle the contribution of each of the transmission modes to viral spread. To this end, we simulated infection dynamics allowing for simultaneous occurrence of cell-free and cell-to-cell transmission in our agent-based model. Hereby, infected cells produce virions that are exported to the extracellular space and diffuse through the simulated space before infecting other cells (see the Materials and Methods). Sampling the number of infected cells and the extracellular viral load at various time points, we then determined the ability of the different ODE models (Equations (1) and (11)) to predict the proportion of cells infected by either cell-free (CF proportion) or cell-to-cell transmission as observed in the ABM-simulations.

**Figure 6:**
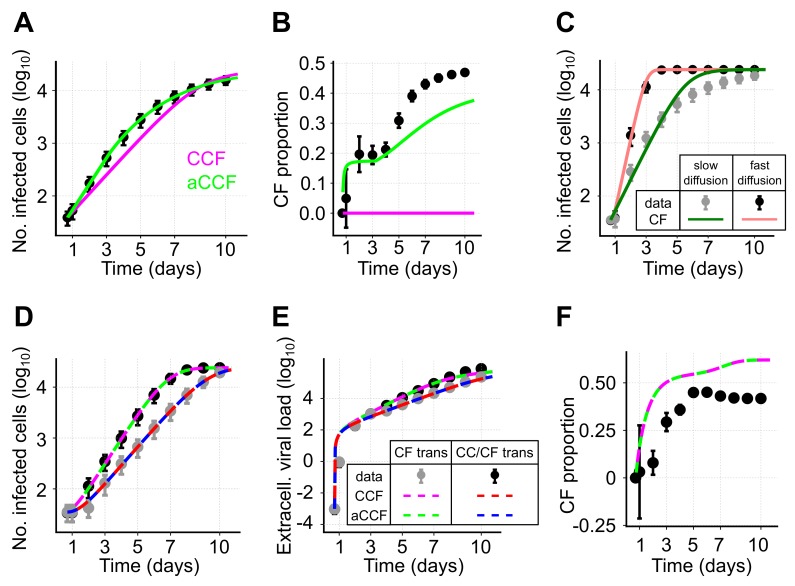
Disentangling viral transmission modes: (**A**,**B**) Simulated data and best model fits for a scenario assuming simultaneous occurrence of both transmission modes given slow diffusion of cell-free virus. Black dots indicate the mean and the 95%-confidence interval of the data obtained from 100 individual simulations runs of the ABM sampled every 24 h. (**A**) Lines show the best fits of a model allowing for both transmission modes without (CCF-model, magenta, Equation (1)) or with adjusted cell-to-cell transmission dynamics (aCCF-model, green, Equation (11)) fitted to the number of infected cells; (**B**) The observed (black dots) and predicted (solid lines) proportion of cells infected by cell-free (CF) transmission dependent on the best fit shown in panel (**A**); (**C**) Simulations of a scenario only allowing for CF-transmission with slow viral diffusion (gray) and fast viral diffusion (black). Lines indicate the best fit of the standard model (Equation (1) with $\beta_{c}=0$) to the data; (**D**–**F**) Similar scenario as in (**A**) with simultaneous occurrence of both transmission modes this time assuming high viral diffusion rates (black, CC/CF trans) and additionally considering a scenario with only CF-transmission (gray, CF trans). Both scenarios are fitted simultaneously. The number of infected cells (**D**), as well as the dynamics of the extracellular viral load (**E**) are shown. (**F**) The observed (black) and predicted proportions of cells infected through CF-transmission in the scenario allowing for both transmission modes. Corresponding parameter estimates for each of the different models are shown in Table 3.

In the first step, we considered a scenario with virions diffusing slowly through the extracellular environment, i.e., considering a low viral diffusion rate. For simplicity, we again neglected cell proliferation and cell death within the ABM-simulations. As before, we observe that our adjusted model considering both transmission modes (aCCF-model, Equation (11) with $\delta_{I}=\delta_{T}=0$) provides a substantially better description of the observed dynamics than the standard model (CCF-model, Equation (1) with $\delta_{I}=\delta_{T}=0$) (Figure 6A, ΔAICc=60, Table 3). Based on the estimates for $\beta_{c}$ and $\beta_{f}$, the CCF-model also predicts that all cells are infected by only one of the two transmission modes. In contrast, the aCCF-model indicates an increasing proportion of cells infected by cell-free transmission, which is comparable to the observations in the simulated data (Figure 6B). However, at later time points, the CF proportion is generally underestimated which indicates an underestimation of the corresponding transmission rate, $\beta_{f}$. Thus, while the standard model is incapable of inferring the contribution of each of the two transmission modes to infection spread, our adjusted model provides appropriate approximations, although it underestimates the CF proportion at later time points.

Identifying the contribution of each of the transmission modes to viral spread could potentially be facilitated if one of the transmission rates is already known, or if it can be quantified based on separate experiments. Assuming that cell-to-cell transmission can be blocked, we simulated infection spread in our ABM only allowing for cell-free transmission. Fitting the standard model of virus dynamics (CF-model, Equation (1), $\beta_{c}=0$) to these data, we observe that a correct quantification of $\beta_{f}$ is impaired under conditions in which the extracellular virus diffuses slowly (Figure 6C). Specifically, in cases of low viral diffusion rates infection tends to spread locally, causing a model assuming mass-action kinetics for infection spread to provide a poor description of the dynamics. This could already be seen for local spread by cell-to-cell transmission (e.g., Figure 4A). However, given fast viral diffusion rates (∼1.7 μm${}^{2}$s${}^{-1}$ resembling those observed for HIV-sized virions diffusing in water [31], Table S1), which are roughly 165-times faster than assumed before, a model describing cell-free transmission by mass-action kinetics would provide a reasonable description of the observed dynamics as the system approaches a well-mixed situation (Figure 6C). Thus, a separate quantification of the rate of cell-free transmission is only appropriate in case of fast viral diffusion rates allowing far-range viral spread.

We therefore tested if the simultaneous analysis of simulated data from systems considering either only cell-free or both transmission modes in case of fast viral diffusion allows us to disentangle the contribution of each of the two transmission modes to infection spread. We found that both models, CCF and aCCF, provide reasonable descriptions of the dynamics for infected cells and extracellular virus (Figure 6D,E). Furthermore, both models lead to similar estimates for the cell-free and cell-to-cell transmission rates, $\beta_{f}$ and $\beta_{c}$, respectively (Figure 6F and Table 3). In this scenario, there is no indication for the necessity of an adjustment term that corrects for a decreasing frequency of CC contributors (ΔAICc=9.5, Table 3). Due to a fast viral diffusion, many new foci are initiated during the time course of infection, which in turn leads to small average focus sizes. Despite their ability to describe the infection kinetics (Figure 6F), both models slightly overestimate the proportion of cells infected by cell-free virus, although capturing the general dynamics (Figure 6D,E).

In summary, population dynamics models are able to provide a rough approximation for the proportion of cells infected by either of the transmission modes if viral diffusion rates allow for far-range viral spread, and in case one of the transmission kinetics can be quantified separately. In the case of high viral diffusion, there is generally no evidence for using the adjusted aCCF-model. However, if CF infection tends to spread locally, i.e., if viral diffusion rates are low, using the adjusted model aCCF-model is recommended as it provides far better approximations than the previously used CCF-model.

## 4. Discussion

Direct cell-to-cell transmission of viral material between infected and uninfected cells has been found to play an important role for various viruses, including hepatitis C virus, influenza and HIV-1 (reviewed in [1]). As cell-to-cell transmission is thought to have the potential to shield the virus from host neutralizing antibodies and other viral clearance mechanisms [2,3,21,32], it has been estimated that this route of transmission is much more efficient than cell-free viral spread [4,5]. However, to reliably infer the contribution of this transmission mode for the spread of infection, appropriate mathematical models that analyze the infection dynamics are essential.

In the current study we show that standard population-based approaches fall short to explain viral spread within spatially defined environments given cell-associated viral spread. In particular for stationary cells, ODE models relying on mass-action kinetics to describe spread of infection by cell-to-cell (CC) transmission do not explain the observed dynamics (Figure 2B). This can be explained by the fact that such models assume that all infected cells are capable of contributing to cell-to-cell spread while the actual proportion is decreasing with increasing focus sizes. Spatial effects limit the proportion of contributors as soon as infected cells are completely surrounded by other infected cells. Models not accounting for this loss underestimate the actual transmission kinetics by overestimating the population of cells driving the spread of infection. Extending previous virus dynamics models by adding a newly developed adjustment term that accounts for this decreasing proportion of CC contributors over time, we were able to correctly describe the underlying dynamics. This novel approach provides more appropriate estimates for the kinetics of cell-to-cell transmission among stationary cells, with estimates for the cell-to-cell transmission rates being roughly 1.5-times larger than estimates relying on the uncorrected models. In this scenario, estimates based on mathematical models that rely on mass-action kinetics only provide a lower bound for the transmission kinetics as they tend to overestimate the cell population contributing to infection. To what extent this also applies for cell-to-cell transmission among motile target cells still has to be investigated. However, it has already been shown that the assumption of a well-mixed situation has to be taken with care if cell-cell interactions take time, and if multiple cells interact simultaneously [33,34,35]. With the observed importance and assumed efficiency of a direct viral transmission pathway between infected and uninfected cells for several viruses, it is more important than ever to correctly account for this mode of transmission when analyzing viral spread.

As appropriate experimental methods to distinguish between cells infected by either route of transmission are lacking, mathematical models currently represent a feasible alternative strategy to estimate the contribution of each of the transmission modes to viral spread. However, disentangling the contribution of cell-free and cell-to-cell transmission to viral spread by population dynamics models in case of their simultaneous occurrence is generally challenging (Figure 6). Our analysis based on simulated data indicates that our extended ODE model provides a better approximation for the proportion of cells infected by each transmission mode compared to previous approaches, when cell-free virus tends to spread locally (Figure 6B). However, an underestimation of the proportion of cells infected by cell-free transmission at later time points is still observed, and is to be expected. Specifically, when viral diffusion rates are low growth patterns are more likely to resemble those relying on cell-to-cell transmission dynamics causing the contribution of cell-free transmission to be underestimated as it cannot be reliably separated from growth of foci by cell-to-cell transmission. Analysis of data from experiments where either cell-free [21] or cell-to-cell transmission [14] is inhibited can be used to quantify one of the transmission kinetics separately. This additional information clearly improves the quantification of the contribution of each of the transmission modes to viral spread. Hereby, the kinetics of cell-free transmission can only be reliably estimated by population-dynamics models if viral diffusion rates allow for far-range viral spread leading to non-local growth patterns. However, even with such prior-knowledge biased estimates can still be obtained as the mean-field approximations by ODE models can only insufficiently account for the effects both transmission modes have on each other (Figure 6F).

For simplicity, our analysis explicitly concentrated on the viral transmission dynamics and neglected infected cell death and the turnover of uninfected target cells in the simulated data and analysis. As such, our approach could be especially relevant for non-cytopathic viruses, such as hepatitis B and C virus, where cell death can be ignored until effective immune responses are induced [36,37]. To which extent target cell turnover, as well as recovery of tissue could affect the dynamics and, thus, the appropriateness of our extension term, remains to be investigated. However, several theoretical studies including target cell dynamics already revealed the limitations of modeling approaches using simple mass-action kinetics in case of spatial effects [24,38]. In ecological and epidemiological studies, it has also been shown that the consideration of space for contact-dependent infection spread has important implications on the epidemiological and evolutionary dynamics [39,40]. Spatial separation of the susceptible population can lead to delayed and more reduced infection kinetics, as well as a higher likelihood of epidemics to go extinct [39]. A number of different modeling frameworks have been developed that study infection spread within populations and that have also been used to determine the effect of space in the context of viral spread within tissues. These modeling frameworks include meta-population models [24], pairwise-approximation or spatial moment equations [41], models based on partial differential equations [31], and even sophisticated agent-based models and cellular automata [22,23]. However, these studies mainly concentrated on the qualitative behavior of the systems due to the general difficulty of fitting them to experimental data. Nevertheless, they already revealed differences in the infection dynamics in comparison to non-spatial models and limitations for their assumptions [24,38,42]. Thus, especially in the context of experimental systems of increasing complexity that mimic natural tissue structures [43,44,45], reliably accounting for space will be important when analyzing viral spread.

Our novel approach represents a first step towards an improved quantification of cell-to-cell transmission dynamics within complex environments using ODE-based virus dynamics models. It is especially relevant for viruses spreading within solid tissues, such as hepatitis C virus within the liver [18,20] or influenza spreading in lung epithelial tissue [23]. Most importantly, the extended model formalism allows for consideration of viral spread on the cellular level, while remaining applicable to the standard population-based experimental data typically available for analysis (e.g., [14]). While individual cell-based approaches, such as agent-based models as presented here and elsewhere [22,46], allow a more detailed description and analysis of the transmission kinetics, the complex data required to utilize such models is less often available, and the computational complexity of the models themselves can be limiting. In combination with the advances in computational methods that allow parameter estimation for such sophisticated models [47], these approaches could enhance the possibility to infer the kinetics and contribution of each of the transmission modes to viral spread for different pathogens and, thus, could provide necessary information for the development of potent antiviral strategies.

## Figures and Tables

**Table 1:** Overview of all different models considered that were applied to the ABM-simulated data. As we always neglect cellular turnover in our simulations to focus on the effect of the spatial conditions on the transmission processes, cell death is always neglected within the model equations, meaning $\delta_{T}=\delta_{I}=0$.

Name	Description	Equation #	Assumptions
CF	cell-free (CF) transmission model	(1)	$\beta_{c}=0$
CC	cell-to-cell (CC) transmission model	(1)	$\beta_{f}=0$
CCF	CF and CC model	(1)	
aCC	adjusted CC model	(11)	$\beta_{f}=0$
aCC-d${}_{I}$	adjusted CC model with delay on infected cells	(11)	$\beta_{f}=0$; $\tau_{I}$ included
aCCF	CF and adjusted CC model	(11)	

**Table 2:** Estimates of the cell-to-cell (CC) transmission dynamics in the simulated data. The CC-, aCC- and aCC-d${}_{I}$ model (see Table 1 for equations) are fitted to the concentration of infected cells obtained for the growth of single and multiple foci. The parameter estimate of the best fit is given with the 95%-confidence interval shown in brackets. Model performance was assessed using the corrected Akaike information criterion (AICc). The difference in the corrected AIC values, ΔAICc, is calculated relative to the AICc of the best fitting model (ΔAICc=0) for single and multiple focus growth, respectively. For the aCC-model, the neighbor distribution was defined by k=6. The parameter θ scales the number of infected cells within the adjustment term and defines a quantity representing irregular focus growth, with θ=1 indicating perfect circular focus growth. For multiple foci, the number of infected cells is divided by ϕ=ψθ, where ψ defines the number of initially infected cells (see also Supplementary Material Text S1). Viral turnover rates, i.e., *c* and ρ, are not taken into account when analyzing only CC-transmission dynamics.

Model:	CC	aCC	$\mathrm{aCC}-d_{I}$
	**Single focus**		
$\beta_{c}$ $\left(\times 10^{-6}\;h^{-1}{\mathrm{cell}}^{-1}\right)$	1.04[1.00,1.07]	1.49[1.47,1.50]	2.04[1.82,2.34]
θ	-	2.7[2.58,2.82]	1.62[1.31,1.94]
*z* (cells)	-	13.6[8.82,17.9]	20.2[12.5,28.6]
τ (hours)			5.71[3.73,7.89]
ΔAICc	823.1	29.2	0
	**Multiple foci**		
$\beta_{c}$ $\left(\times 10^{-6}\;h^{-1}{\mathrm{cell}}^{-1}\right)$	1.13[1.07,1.18]	1.72[1.70,1.74]	1.72[1.70,1.79]
θ	-	1.78[1.70,1.86]	1.78[1.66,1.86]
*z* (cells)	-	2.19(0,6.61]	2.09(0,6.31]
τ (hours)			$1.65\times 10^{-5}\;\left(0,0.664\right]$
ΔAICc	92.88	0	7.33

**Table 3:** Estimated transmission rates for model predictions fitted to simulated data allowing simultaneous transmission dynamics. The transmission rates describing cell-free, $\beta_{f}$, and cell-to-cell transmission, $\beta_{c}$, are denoted in $\times 10^{-9}h^{-1}{\mathrm{cell}}^{-1}$ and $\times 10^{-8}h^{-1}$, respectively. For the simultaneous fit of both transmission modes to the number of infected cells without separate quantification of cell-free transmission (Figure 6A) no reasonable values for $\beta_{f}$ can be obtained, i.e., $\beta_{f}$ is not identifiable (ni). Confidence intervals are calculated from 2 × $10^{3}$ (CC/CF) and $10^{3}$ (CC/CF + CF) individual fits using random starting conditions and considering all parameter combinations within a range of 3.84 of the best fit likelihood. The difference in the corrected AIC values, ΔAICc, is calculated relative to the AICc of the best fitting model for each scenario, i.e., ΔAICc=0.

Scenario:	Both Transmission Modes		Both Transmission Modes + Only CF	
	(CC/CF)		(CC/CF + CF)	
Model:	CCF	aCCF	CCF	aCCF
$\beta_{c}$	2.37 (0, 2.41]	2.74 [2.67, 3.00]	1.52 [1.50, 1.54]	1.52 [1.50, 1.63]
$\beta_{f}$	ni	ni	4.30 [3.65, 5.20]	4.30 [3.67, 4.80]
ΔAICc	60	0	0	9.5

## Supplementary Materials

The following are available online at http://www.mdpi.com/1999-4915/10/4/200/s1: Figure S1: Profile likelihood analysis for parameter estimates considering single focus growth, Table S1: Parameters used for simulating viral spread in the agent-based model, Table S2: Estimates for the aCC-model given multiple foci using different fixed values for the assumed number of neighbors per cell, *k*, Text S1: Mathematical derivation of a continuous adjustment term for cells contributing to cell-to-cell transmission.

## Abbreviations

The following abbreviations are used in this manuscript:

CC	Cell-to-cell
CF	Cell-free

## References

[1] Sattentau Q. (2008). Avoiding the void: Cell-to-cell spread of human viruses. Nat. Rev. Microbiol..

[2] Sigal A., Kim J.T., Balazs A.B., Dekel E., Mayo A., Milo R., Baltimore D. (2011). Cell-to-cell spread of HIV permits ongoing replication despite antiretroviral therapy. Nature.

[3] Brimacombe C.L., Grove J., Meredith L.W., Hu K., Syder A.J., Flores M.V., Timpe J.M., Krieger S.E., Baumert T.F., Tellinghuisen T.L. (2011). Neutralizing antibody-resistant hepatitis C virus cell-to-cell transmission. J. Virol..

[4] Chen P., Hubner W., Spinelli M.A., Chen B.K. (2007). Predominant mode of human immunodeficiency virus transfer between T cells is mediated by sustained Env-dependent neutralization-resistant virological synapses. J. Virol..

[5] Sato H., Orenstein J., Dimitrov D., Martin M. (1992). Cell-to-cell spread of HIV-1 occurs within minutes and may not involve the participation of virus particles. Virology.

[6] Abela I.A., Berlinger L., Schanz M., Reynell L., Gunthard H.F., Rusert P., Trkola A. (2012). Cell-cell transmission enables HIV-1 to evade inhibition by potent CD4bs directed antibodies. PLoS Pathog..

[7] Barretto N., Sainz B., Hussain S., Uprichard S.L. (2014). Determining the involvement and therapeutic implications of host cellular factors in hepatitis C virus cell-to-cell spread. J. Virol..

[8] Perelson A.S. (2002). Modelling viral and immune system dynamics. Nat. Rev. Immunol..

[9] Canini L., Perelson A.S. (2014). Viral kinetic modeling: State of the art. J. Pharmacokinet. Pharmacodyn..

[10] Graw F., Perelson A.S. (2016). Modeling Viral Spread. Annu. Rev. Virol..

[11] Perelson A.S., Neumann A.U., Markowitz M., Leonard J.M., Ho D.D. (1996). HIV-1 dynamics in vivo: Virion clearance rate, infected cell life-span, and viral generation time. Science.

[12] Neumann A.U., Lam N.P., Dahari H., Gretch D.R., Wiley T.E., Layden T.J., Perelson A.S. (1998). Hepatitis C viral dynamics in vivo and the antiviral efficacy of interferon-alpha therapy. Science.

[13] Culshaw R.V., Ruan S., Webb G. (2003). A mathematical model of cell-to-cell spread of HIV-1 that includes a time delay. J. Math. Biol..

[14] Iwami S., Takeuchi J.S., Nakaoka S., Mammano F., Clavel F., Inaba H., Kobayashi T., Misawa N., Aihara K., Koyanagi Y. (2015). Cell-to-cell infection by HIV contributes over half of virus infection. eLife.

[15] Zhang C., Zhou S., Groppelli E., Pellegrino P., Williams I., Borrow P., Chain B.M., Jolly C. (2015). Hybrid spreading mechanisms and T cell activation shape the dynamics of HIV-1 infection. PLoS Comput. Biol..

[16] Komarova N.L., Levy D.N., Wodarz D. (2013). Synaptic transmission and the susceptibility of HIV infection to anti-viral drugs. Sci. Rep..

[17] Komarova N.L., Wodarz D. (2013). Virus dynamics in the presence of synaptic transmission. Math. Biosci..

[18] Kandathil A.J., Graw F., Quinn J., Hwang H.S., Torbenson M., Perelson A.S., Ray S.C., Thomas D.L., Ribeiro R.M., Balagopal A. (2013). Use of laser capture microdissection to map hepatitis C virus-positive hepatocytes in human liver. Gastroenterology.

[19] Graw F., Balagopal A., Kandathil A.J., Ray S.C., Thomas D.L., Ribeiro R.M., Perelson A.S. (2014). Inferring viral dynamics in chronically HCV infected patients from the spatial distribution of infected hepatocytes. PLoS Comput. Biol..

[20] Wieland S., Makowska Z., Campana B., Calabrese D., Dill M.T., Chung J., Chisari F.V., Heim M.H. (2014). Simultaneous detection of hepatitis C virus and interferon stimulated gene expression in infected human liver. Hepatology.

[21] Graw F., Martin D.N., Perelson A.S., Uprichard S.L., Dahari H. (2015). Quantification of Hepatitis C Virus Cell-to-Cell Spread Using a Stochastic Modeling Approach. J. Virol..

[22] Bauer A.L., Beauchemin C.A., Perelson A.S. (2009). Agent-based modeling of host-pathogen systems: The successes and challenges. Inform. Sci..

[23] Beauchemin C., Samuel J., Tuszynski J. (2005). A simple cellular automaton model for influenza A viral infections. J. Theor. Biol..

[24] Funk G.A., Jansen V.A., Bonhoeffer S., Killingback T. (2005). Spatial models of virus-immune dynamics. J. Theor. Biol..

[25] Aunins T.R., Marsh K.A., Subramanya G., Uprichard S.L., Perelson A.S., Chatterjee A. (2018). Intracellular hepatitis C modeling predicts infection dynamics and viral protein mechanisms. J. Virol..

[26] R Core Team (2016). R: A Language and Environment for Statistical Computing.

[27] Raue A., Kreutz C., Maiwald T., Bachmann J., Schilling M., Klingmüller U., Timmer J. (2009). Structural and practical identifiability analysis of partially observed dynamical models by exploiting the profile likelihood. Bioinformatics.

[28] Burnham K.P., Anderson D.R. (2002). Model Selection and Multinomal Inference: A Practical Information-Theoretic Approach.

[29] Sabahi A., Marsh K.A., Dahari H., Corcoran P., Lamora J.M., Yu X., Garry R.F., Uprichard S.L. (2010). The rate of hepatitis C virus infection initiation in vitro is directly related to particle density. Virology.

[30] Barretto N., Uprichard S.L. (2014). Hepatitis C virus Cell-to-cell Spread Assay. Bio-Protocol.

[31] Strain M.C., Richman D.D., Wong J.K., Levine H. (2002). Spatiotemporal dynamics of HIV propagation. J. Theor. Biol..

[32] Reh L., Magnus C., Schanz M., Weber J., Uhr T., Rusert P., Trkola A. (2015). Capacity of Broadly Neutralizing Antibodies to Inhibit HIV-1 Cell-Cell Transmission Is Strain- and Epitope-Dependent. PLoS Pathog..

[33] Graw F., Regoes R.R. (2009). Investigating CTL Mediated Killing with a 3D Cellular Automaton. PLOS Comput. Biol..

[34] Gadhamsetty S., Marée A.F.M., Beltman J.B., de Boer R.J. (2017). A General Functional Response of Cytotoxic T Lymphocyte-Mediated Killing of Target Cells. Biophys. J..

[35] Pilyugin S.S., Antia R. (2000). Modeling immune responses with handling time. Bull. Math. Biol..

[36] Dahari H., Major M., Zhang X., Mihalik K., Rice C.M., Perelson A.S., Feinstone S.M., Neumann A.U. (2005). Mathematical modeling of primary hepatitis C infection: Noncytolytic clearance and early blockage of virion production. Gastroenterology.

[37] Cheng X., Xia Y., Serti E., Block P.D., Chung M., Chayama K., Rehermann B., Liang T.J. (2017). Hepatitis B virus evades innate immunity of hepatocytes but activates cytokine production by macrophages. Hepatology.

[38] Beauchemin C. (2006). Probing the effects of the well-mixed assumption on viral infection dynamics. J. Theor. Biol..

[39] Keeling M.J., Rohani P. (2008). Modeling Infectious Diseases in Humans and Animals.

[40] Lion S., Baalen M.V. (2008). Self-structuring in spatial evolutionary ecology. Ecol. Lett..

[41] Lion S. (2016). Moment equations in spatial evolutionary ecology. J. Theor. Biol..

[42] Holder B.P., Liao L.E., Simon P., Boivin G., Beauchemin C.A. (2011). Design considerations in building in silico equivalents of common experimental influenza virus assays. Autoimmunity.

[43] Fackler O.T., Murooka T.T., Imle A., Mempel T.R. (2014). Adding new dimensions: Towards an integrative understanding of HIV-1 spread. Nat. Rev. Microbiol..

[44] Glushakova S., Baibakov B., Margolis L.B., Zimmerberg J. (1995). Infection of human tonsil histocultures: A model for HIV pathogenesis. Nat. Med..

[45] Baker B.M., Chen C.S. (2012). Deconstructing the third dimension: How 3D culture microenvironments alter cellular cues. J. Cell. Sci..

[46] Beltman J.B., Maree A.F., Lynch J.N., Miller M.J., de Boer R.J. (2007). Lymph node topology dictates T cell migration behavior. J. Exp. Med..

[47] Jagiella N., Rickert D., Theis F.J., Hasenauer J. (2017). Parallelization and High-Performance Computing Enables Automated Statistical Inference of Multi-scale Models. Cell Syst..

