# Supplementary material for: Accounting for Space — Quantification of Cell-To-Cell Transmission Kinetics Using Virus Dynamics Models
Source: Viruses. 2018 Apr 17;10(4):200. doi: 10.3390/v10040200 (PMC5923494; doi:10.3390/v10040200)
Supplement: Supplementary file 1 [file viruses-10-00200-s001.zip › SI_files/S1_Figure_pkumbergerFINAL.pdf]

Figure S1: Profile likelihood analysis for parameters estimates that characterize the adjustment term considering single focus growth.

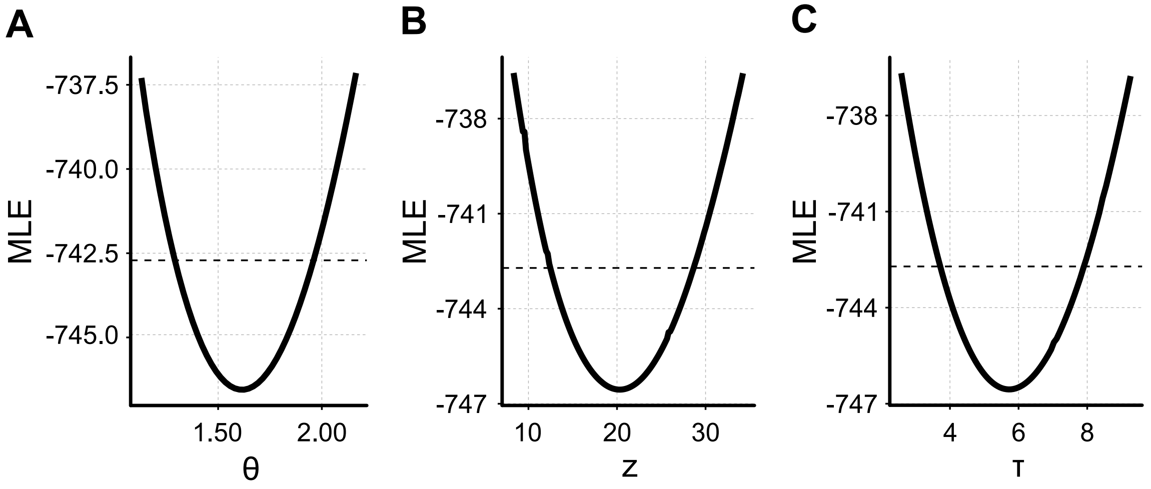

All profiles indicate the identifiability of the parameters based on the simulated data. **(A)** Profile likelihood for the parameter $\theta := \phi\psi$, where ψ defines a measure of non-circular focus shapes and ϕ the number of initially seeded foci (see also *Supplementary Material* Text S1). **(B)** Profile likelihood for the interval length, z, for which the smooth connection term, f_2 of our adjustment is applicable. **(C)** Profile likelihood for the time delay, τ, for infected cells to become infectious.
