# Supplementary material for: Accounting for Space — Quantification of Cell-To-Cell Transmission Kinetics Using Virus Dynamics Models
Source: Viruses. 2018 Apr 17;10(4):200. doi: 10.3390/v10040200 (PMC5923494; doi:10.3390/v10040200)
Supplement: Supplementary file 1 [file viruses-10-00200-s001.zip › SI_files/S1_Table_pkumbergerFINAL.pdf]

Table S1: Parameters used in the agent-based model to simulate viral spread.

Parameter	Description	Unit	Value
cells	Total nbr. of cells per well	cells	24031
t_{sim}	Simulation time	h	240
t_{init}	Initialization period	h^{-1}	17
α_{init}	Infection probability during initialization period	h^{-1}	6×10^{-3}
c_{init}	Average number of cells infected during initialization period	cells	35
R_0	Initial positive-strand RNA at infection	RNA	1
α	Maximal RNA production rate	h^{-1}	0.446
ρ	Export rate for enveloped virus	h^{-1}	2.01×10^{-3}
γ	Degradation rate of intracellular RNA	h^{-1}	0.2
R_{cap}	Capacity of intracellular RNA per infected cell	RNA	2369
s_{f}	scaling factor for CF transmission probability	$(h\times \text{ intrac. RNA})^{-1}$	49.2
s_{c}	scaling factor for CC transmission probability	$(h\times \text{ extrac. Virus})^{-1}$	1680
f_{inf}	Fraction of infectious virus per intracellular RNA		1×10^{-3}
m	Diffusion parameter (slow)	$\mu m^2\ h^{-1}$	3.75×10^{1}
m	Diffusion parameter (fast)	$\mu m^2\ h^{-1}$	6.25×10^{3}
c	Loss of infectivity of extracellular virus	h^{-1}	6.34×10^{-2}

For simulations shown in Figure 6(D-F), values of $s_{\mathrm{c}} = 1 \times 10^{-5}$ $(\text{min}\times \text{ intrac. RNA})^{-1}$ and $s_{\mathrm{f}} = 1 \times 10^{-4}$ $(\text{min}\times \text{ extrac. Virus})^{-1}$ were used.
