# Supplementary material for: Accounting for Space — Quantification of Cell-To-Cell Transmission Kinetics Using Virus Dynamics Models
Source: Viruses. 2018 Apr 17;10(4):200. doi: 10.3390/v10040200 (PMC5923494; doi:10.3390/v10040200)
Supplement: Supplementary file 1 [file viruses-10-00200-s001.zip › SI_files/S1_Text_pkumbergerFINAL.pdf]

Text S1: Mathematical derivation of a continuous adjustment term for cells contributing to cell-to-cell transmission

Assuming regular growth of a focus of infected cells in a 2D monolayer of cells, we developed the adjustment term, $f_c(k, I)$, by

$$f_c(k, I) = \frac{\sqrt{k^2 + 8kI - 8k} - k}{2I}. \quad (1)$$

It describes the proportion of cells that contribute to cell-to-cell transmission as a continuous function of the assumed number of neighbors per cell, k, and the total number of infected cells in the focus, I (see also Eq. (9) in the main manuscript).

As indicated in the manuscript, the general function to approximate the frequency of CC contributors (Eq. (1)) is only valid for large foci sizes. For foci sizes smaller than the assumed number of neighbors, $I < k$, all cells would still be able to contribute to cell-to-cell transmission, thus, $f_1 = 1$. In order to allow for a smooth adjustment term correcting Eq. (1) for small foci sizes, we derived a transition term f_2 that connects $f_1 = 1$ and f_3 defined by Eq. (1) (see also Fig. 3 in the main manuscript). The term f_2 is defined as a smooth polynomial of third degree given by

$$f_{c,2}(x) = a(x-k)^3 + b(x-k)^2 + c(x-k) + d \quad (2)$$

with the parameters a, b, c and d determining the coefficients of the polynomial and k the number of assumed neighbors. In order to allow for a smooth transition from f_1 to f_3, the term f_2 needs to satisfy the following conditions:

1. $f_2(k) = f_1(0) = 1$
2. $\frac{df_2}{dI}(k) = \frac{df_1}{dI}(0) = 0$
3. $f_2(k+z) \stackrel{!}{=} f_3(k+z) := J_1$
4. $\frac{df_2}{dI}(k+z) \stackrel{!}{=} \frac{df_3}{dI}(k+z) := J_2$

Conditions 1 and 2 ensure a continuous connection between the first and second part of the adjustment term at position k, i.e. f_1 and f_2, respectively. Similarly, the two other conditions ensure a continuous connection for the second and third part at a total number of $k + z$ cells in the foci, with $z \in \mathbb{N}$. Hereby, z defines the number of infected cells for which the transition term applies. Solving these conditions using $f_1 = 1$, Eq. (1) and Eq. (2), we obtain the following solutions for the coefficients of the polynomial describing f_2:

$$a = \frac{J_2 z + 2 - 2J_1}{z^3}$$
$$b = \frac{3J_1 - J_2 z - 3}{z^2}$$
$$c = 0$$
$$d = 1$$

Hereby, J_1 and J_2 depend on the number of neighbors and define the value of f_2 and f_3 at position $k + z$, respectively. These terms are defined by

$$J_1 = \frac{\sqrt{9k^2 + 8kz - 8k} - k}{2(k + z)}$$
$$J_2 = \frac{k\left(\sqrt{9k^2 + 8zk - 8k} - 5k - 4z + 8\right)}{2(k + z)^2\sqrt{9k^2 + 8kz - 8k}}$$

calculated by Eq. (7) given in the manuscript.

The combined adjustment term, $f_c(I)$, is a measure for the proportion of CC contributors during the growth of an average focus dependent on the total number of infected cells within a focus. The derivation is based on the assumption that each focus follows a circular growth pattern (compare Fig. 3A in the manuscript). To account for non-circular focus growth, we introduced a parameter θ, scaling the number of infected cells in the adjustment term, i.e, $f_c(I/\theta)$. A value of $\theta = 1$ indicates perfect circular growth, while larger values correspond to non-regular focus growth. For multiple foci, the number of infected cells has to be divided by $\phi := \psi\theta$, as described in the derivation of

the adjustment term in the main text, where ψ defines the number of initially infected cells. The parameter θ is estimated when fitting the model to data.
