# Supplementary material for: Accounting for Space — Quantification of Cell-To-Cell Transmission Kinetics Using Virus Dynamics Models
Source: Viruses. 2018 Apr 17;10(4):200. doi: 10.3390/v10040200 (PMC5923494; doi:10.3390/v10040200)
Supplement: Supplementary file 1 [file viruses-10-00200-s001.zip › SI_files/S2_Table_pkumbergerFINAL.pdf.pdf]

Table S2: Estimates for the aCC-model given multiple foci using different fixed values for the assumed number of neighbors per cell, k.

**Parameter**	$k = 4$	$\mathbf{k = 6}$	$k = 8$	$k = 15$
β_c ($\times 10^{-6}\text{h}^{-1}\text{cell}^{-1}$)	1.72 $[1.70, 1.73]$	1.72 $[1.70, 1.74]$	1.72 $[1.70, 1.73]$	1.71 $[1.70, 1.72]$
θ	2.73$[2.66, 2.78]$	1.78 $[1.70, 1.86]$	1.32 $[1.29, 1.37]$	0.69 $[0.67, 0.71]$
z (cells)	2.44$[0.22, 4.80]$	2.19 $(0, 6.61]$	1.12 $(0, 8.55]$	10^{-5} $(0, 15.05]$
AICc	75.7	75.3	75.2	75.6

Data were simulated using $k = 6$. Numbers in brackets represent 95%-confidence intervals for estimates based on 10^4 individual fits. Corrected AIC-values indicate model performance. Parameters include the cell-to-cell transmission rate, β_c, the number of infected cells over which the transition term for the smooth approximation spans, z, and the parameter θ scaling for irregular foci growth (see also Text S1 for a detailed explanation of the parameters). The results show that the estimates for the cell-to-cell transmission rate β_c are robust for deviations in the assumed underlying neighbor-distribution k.
